# Predictors of Functional Outcome in a Cohort of Hispanic Patients Using Exoskeleton Rehabilitation for Cerebrovascular Accidents and Traumatic Brain Injury

**DOI:** 10.3389/fnbot.2021.682156

**Published:** 2021-06-10

**Authors:** Lisa R. Treviño, Peter Roberge, Michael E. Auer, Angela Morales, Annelyn Torres-Reveron

**Affiliations:** ^1^DHR Health Institute for Research and Development, Edinburg, TX, United States; ^2^The DHR Health Rehabilitation Hospital, Edinburg, TX, United States

**Keywords:** trauma, injury, minorities, gait, diabetes

## Abstract

Traumatic brain injury (TBI) and cerebrovascular accidents (CVA) are two of the leading causes of disability in the United States. Robotic exoskeletons (RE) have been approved for rehabilitation by the Federal Drug Administration (FDA) for use after a CVA, and recently received approval for use in patients with TBI. The aim of the study was to determine which factors predict the improvement in functional independence measure (FIM) score after using RE rehabilitation in a population of patients with CVA or TBI. We carried out a retrospective chart-review analysis of the use of the RE (Ekso® GT) in the rehabilitation of patients with TBI and CVA using data from a single, private rehabilitation hospital for patients admitted and discharged between 01/01/2017 and 04/30/2020. From the medical records, we collected presentation date, Glasgow Coma Scale score (GCS) on the date of injury, rehabilitation start date, age, diabetes status on presentation (Yes or No), injury category (TBI or CVA), and both admission and discharge FIM scores. Matching algorithms resulted in one TBI patient matched to three CVA patients resulting in a sample size of 36. The diabetic and non-diabetic populations showed significant differences between age and days from injury to the start of rehabilitation. A multivariate linear regression assessed predictors for discharge motor FIM and found admission motor FIM score and total RE steps to be statistically significant predictors. For each point scored higher on the admission motor FIM the discharge FIM was increased by 1.19 FIM points, and for each 1,000 steps taken in the RE, the discharge motor FIM increased by three points. The type of acquired brain injury (CVA or TBI) was not found to affect functional outcome. The presented results show that key clinic-biologic factors including diabetic status, together with start to rehabilitation play key roles in discharge FIM scores for patients using RE.

**Clinical Trial Registration**: ClinicalTrials.gov, NCT04465019

## Introduction

The use of robotics in rehabilitation medicine has become more popular due to the advantages it offers to the patients and the providers. Wearable robotic exoskeletons (REs) are approved by the Federal Drug Administration (FDA) for rehabilitation following spinal cord injury, CVA (cerebrovascular accident or stroke), and most recently acquired brain injury, inclusive of Traumatic Brain Injury (TBI, Globe Newswire, 2020). In addition, the RE can assist in repetitive task completion in increased doses (e.g., more steps per training). However, patients must meet strict requirements for RE use to ensure their safety (Palermo et al., 2017). All available REs currently in the market have weight and height requirements. Similarly, bone mineral density is a metric for eligibility to reduce fracture risk while using the device (Asselin et al., 2016). Provider recommendations and patient preferences also affect the decision to incorporate a RE in a patient rehabilitation program. While some studies in patients with spinal cord injury and CVA demonstrate benefits from the use of REs, additional evidence for disorders that cause gait and mobility problems such as traumatic brain injury (TBI) are still necessary (Hesse and Werner, 2009; Dijkers et al., 2019). Hence small studies with limited power, as presented herein, are a step forward in evidence toward generating future hypotheses and advancing the field.

The beneficial use of RE following a CVA has been established in the field. The first studies that examined the acceptance of robotics in patients after CVA started in the nineties (Dijkers et al., 1991). Consensus suggests that robotic-assisted gait training in patients after suffering a CVA is more likely to recover independent walking than those who did not use the robotic devices (Mehrholz et al., 2017; Bruni et al., 2018; Nolan et al., 2020). Studies also suggest that the use of robotics in rehabilitation could result in motor skills improvement and transfer into other daily living domains that require similar activities (Fasoli and Adans-Dester, 2019), hence improving cognitive domains. The time from CVA occurrence to the initiation of rehabilitation is critical for determining the patient's functional recovery degree. Multiple studies suggest that early initiation of rehabilitation results in improved long-term outcomes since most of the patient's recovery after a CVA occurs during the initial first 6 months (Kwakkel et al., 2004; Langhorne et al., 2011). Of importance in our patient population is the over indexing in Type 2 diabetes. Prior studies have identified diabetes as a predictive factor for patients' functional progress after a CVA, independent from other comorbidities including cardiovascular disease (Tziomalos, 2014; Wang et al., 2014). Moreover, patients with type 2 diabetes have twice the risk of suffering from an ischemic CVA than non-diabetics (Sarwar N, 2010) rendering diabetes an important variable in CVA rehabilitation.

Motor and ambulation deficits, which may resemble those presented in CVA, are frequently observed in patients with severe to moderate TBI (Williams et al., 2015). According to the Centers for Disease Control, ~3.2–5.3 million people are living with a TBI-related disability in the United States (Centers for Disease Control Prevention, 2019), constituting one of the leading causes of disability across the nation. TBIs are most frequently observed after motor vehicle accidents and falls (Centers for Disease Control and Prevention, 2015; National Spinal Cord Injury Statistical Center, 2019). Mortality due to TBI has significantly decreased in the United States (from 2006 to 2014, Centers for Disease Control and Prevention, 2015) suggestive of the positive impact from rehabilitation services. Similar to CVA, early rehabilitation intervention following TBI has been shown to produce improvements in post-TBI symptomathology (Parrington et al., 2020). Unfortunately, the data on the use of RE on patients with TBI is limited due to its later approval by the FDA for this condition, as compared to CVA. A recent case report of a young adult who suffered TBI, which resulted in right-side hemiplegia, revealed that RE's use during a 4-week inpatient program significantly improved motor functional independence measure (FIM) score from admission to discharge (Nolan et al., 2018). Moreover, using the RE in a patient with TBI showed increased brain cortical activity at the prefrontal, premotor, and motor cortices at follow up compared to no-RE walking (Karunakaran et al., 2020), suggesting that robotic-assisted gait may produce different cortical brain mechanisms activation.

The United States' Rio Grande Valley, along the Texas-Mexico border, is comprised of ~90% Hispanic ethnicity and 90% Mexican descent. This region is characterized by socioeconomic factors that have been demonstrated to affect clinical outcomes. Such factors include non-insured and underinsured status, a tremendous shortage in primary care providers (medically underserved), and over 30% of the population living in poverty (UT Health–School of Public Health Brownsville, 2018). Thirty percent of the Rio Grande Valley residents have diabetes, compared to 12.3% nationwide (Millard et al., 2017), rendering this region a unique site to study the impact of this over prevalent disease on CVA and TBI rehabilitation outcomes.

Inspired by the unique characteristics of our patient population, this study's main goal was to determine which factors predict the improvement in functional independence measure (FIM) score after using RE rehabilitation in a population of patients with CVA or TBI, with the goal of producing preliminary data that could inform future implementation and design of larger clinical trials. Within the model, we included Type 2 diabetes as a variable and other previously reported predictive factors for functional improvement in rehabilitation after CVA (Wang et al., 2014). The model was based on the rationale that TBI and CVA are both acquired brain injuries that produce related neurofunctional impairments (Giles, 2010; Capizzi et al., 2020), both of which could benefit from RE rehabilitation training. The rationale for comparing TBI and CVA is based on a previous study showing very similar linear improvements in motor functioning during conventional rehabilitation (Bode and Heinemann, 2002). Therefore, determining if RE rehabilitation modifies the functional progression in TBI as compared to CVA has the potential of increasing its use for TBI and expand the research done in this area.

## Materials and Methods

### Study Design and Setting

This is an observational, retrospective, chart review cohort study of patients from a private single rehabilitation hospital. Study approval was granted by the local Institutional Review Board and conformed to the Declaration of Helsinki and the US Federal Policy for the Protection of Human Subjects. Due to the retrospective nature of the study, a full waiver of authorization under the Health Insurance Portability and Accountability Act (1996) was submitted by the study team and approved by the Institutional Review Board.

### Participants

Patients who sustained either a TBI or a CVA and used a RE device during their rehabilitation treatment from 01/01/2017 to 04/30/2020 were included in this study. Patients were excluded if their injury was not diagnosed as a CVA or TBI in their electronic medical record. The registered nurse at the rehabilitation facility identified patients based on the inclusion criteria, and this was verified by the doctor in physical therapy who oversees the rehabilitation hospital patient population. Approval and implementation of the RE device at the health system commenced in 2016 for patients that suffered a CVA or TBI. The main requirements for the use of the RE device during rehabilitation included a height range between 1.56 and 1.9 m, weight less than or equal to 100 kg, stable blood pressure (minimal postural changes in blood pressure), the ability to follow basic commands, and normal range of motion in hips, knees, and ankles.

### Variables

For each patient, electronic medical records were reviewed for injury date, Glasgow Coma Scale score (GCS) on presentation, rehabilitation start date, age, diabetes status on presentation (Yes or No), injury category (TBI or CVA), and both admission and discharge functional independence measure (FIM). The change in motor FIM score from admission to discharge was used as the primary outcome for this study. The FIM score evaluates the ability of the patient in day-to-day functions within five major categories: self-care, sphincter control, transfers, locomotion, communication and social cognition (Linacre et al., 1994). The FIM motor component groups 13 of the tasks as follows: self-care (eating, grooming, bathing, dressing—upper body, dressing—lower body, toileting), sphincter control (bladder management, bowel management), transfers from bed/chair/wheelchair, toilet, tub/shower, and locomotion (walk, stairs). Ethnicity was self-reported but not considered as a covariate since the vast majority of our cohort was of Hispanic origin. The overrepresentation of Hispanics/Latinos in the current cohort responds to the demographic distribution of the area the health system serves.

### Matched Participants

**Figure 1:**
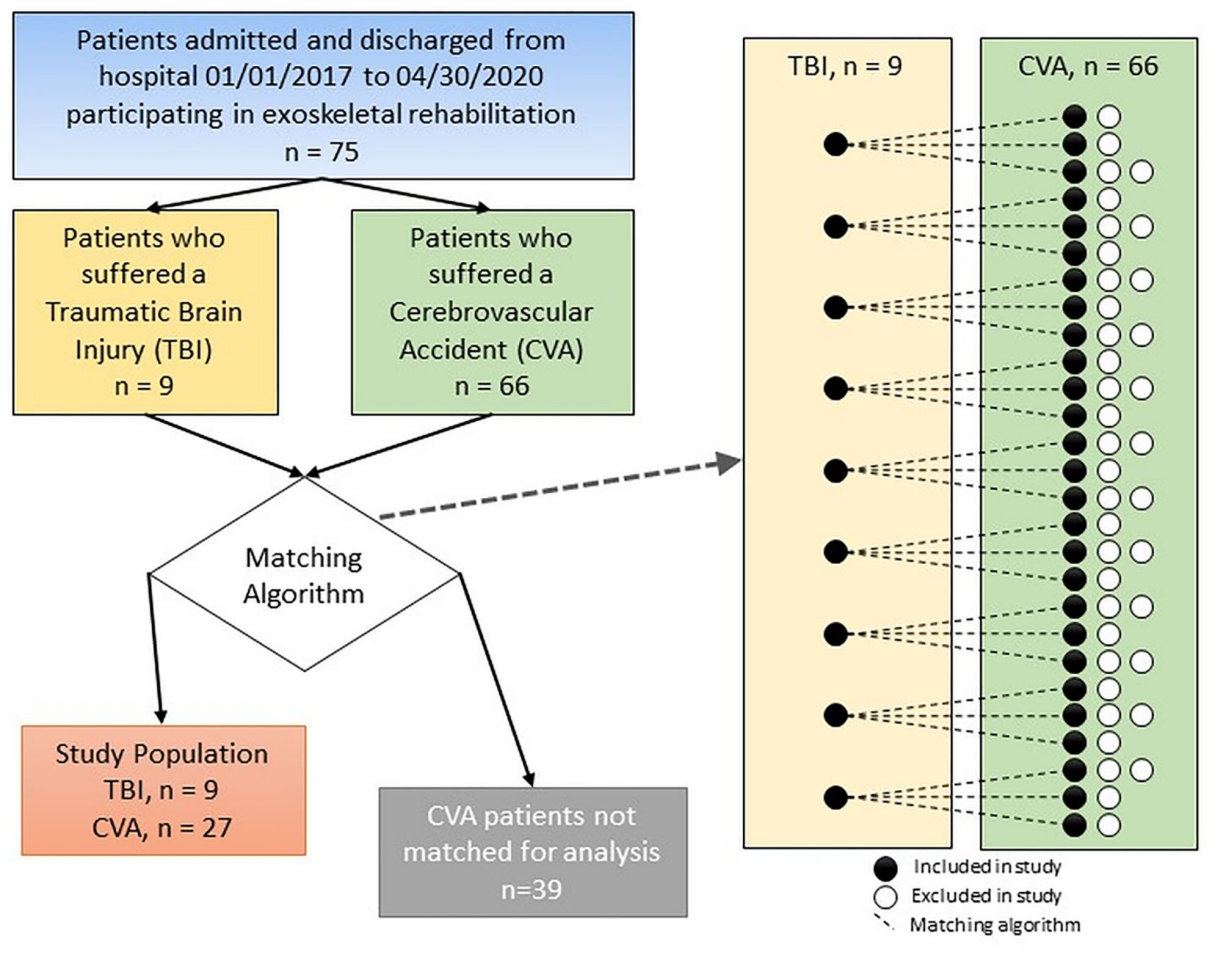
Diagram of the method used for comparing patients in the CVA and TBI groups with the final number of subjects included in each group.

Due to the limited number of TBI cases in the cohort, we decided to use a matching design to control for differences in covariates like age and gender of the patients in our study. The CVA patients were matched to the nine TBI patients algorithmically. A custom R program iteratively calculated the Euclidean distance between TBI and CVA independent variables, then matched patients based on minimum values (R Core Team, 2020). Each TBI patient matched to a CVA patient before starting another iteration of the algorithm (see Figure 1). Power analysis revealed an optimal configuration at three CVA patients matched to each TBI patient. Equalizing the number of matches between TBI patients (each TBI patient matched to three CVA patients) helps reduce algorithmic bias by not over-representing any of the TBI patient's features in the dataset. Matching criteria were days between symptom onset and rehab start, GCS, gender, diabetes status, and age. Patients were not matched on any post-admission variables. Distance was calculated and then ranked for each CVA-TBI patient pair. Ties in ranking were assigned randomly.

### Data Sources/Measurements

All information presented in this study was part of the patient's standard of care. No additional intervention or data collection instrument was implemented. The standard of care of the patient was as follows: all potentially eligible candidates were first identified by a registered nurse and then cleared by the physician with subsequent patient education regarding the use of the RE. For the use of the RE during the rehabilitation process, signed informed consent was obtained from either the patient or designated family member, depending on the status of the patient. Data obtained during the active use of the RE was monitored by an in-device screen (time active, steps taken, etc.) and subsequently recorded in each patient's paper chart. This paper was then scanned and uploaded into the patient's electronic chart under his/her progress notes. The therapy technicians helped in the data recording process. All available data related to RE use for CVA and TBI at our facility within the period of investigation were included in the current study. All patients were also receiving conventional physical therapy during their rehabilitation process, as recommended by the physician and the established standard of care practices at the institution.

### Reduction of Bias

To minimize selection bias in the current study, we used all patients that were clinically determined by the attending physician to meet the criteria for RE rehabilitation. Patients were included in the chart review regardless of the number of sessions of exoskeleton use. All therapist and nurses at our facility have been certified to use the FIM assessment tool and the data is entered using the Uniform Data System for Medical Rehabilitation software (Uniform Data system for Medical Rehabilitation, 2020) integrated within the hospital medical record platform. The main data extractor in the study (A.M.) is the site expert for data management within the Uniform Data System. Quality of the data was verified by the lead doctor in Physical Therapy (M.E.A.). None of the patients that used RE in rehabilitation had missing data during the time period used for the current study and all patients in the cohort completed the recommended therapeutic program and were successfully discharged from the hospital.

### Exoskeleton Use Protocol

The RE was operated by a Licensed Physical Therapist who was certified to control the device. The rehabilitation facility used the Ekso GT® robotic exoskeleton (Ekso Bionics, Richmond, CA), which is only approved to be used in a clinical setting. For the purposes of this study, four different modes were utilized to engage and challenge the patients: Pre-Gait, First Step, Pro Step Plus, and 2 Free. Pre-Gait Mode focused on the following metrics to facilitate movement and active participation: bilateral weight shifting in standing with biofeedback, mini squats, and stationary unilateral lower extremity advancement. The First Step mode allowed the therapist to be in full control of all movement. The therapist triggered the initiation and execution of each step reciprocally with the push of a button. In the Pro Step Plus mode, there was an appropriate weight shift either done independently by the patient or facilitated by the therapeutic handling of the physical therapist. The patient was given the opportunity to initiate each step; the device completed any incomplete steps. The most advanced mode is called “2-Free.” This mode was used to challenge the patient across the continuum as they progress. The patient was responsible for initiating and executing each step. The therapist programmed resistance unilaterally or bilaterally to the lower extremities to increase the demand for the activity.

### Statistical Methods

Data analysis was preformed using R version 4.3 (R Core Team, 2020). Data were summarized using summary statistics and frequency tables. Inspection of scatterplots revealed linear relationships between independent and dependent variables. Zero-order Pearson correlation coefficients were examined and subsequently tested for significance. Diabetic status and acquired brain injury were coded as categorical dummy variables (absence or presence of diabetes status; having either CVA or TBI). Backward, stepwise, multivariate linear regression was used to evaluate the associations of each of the outcome variables with age, sex, days between injury onset and rehabilitation start, and the injury groups (CVA and TBI). *T*-tests assessed significance of the predictors within the regression. Second order variables with *p*-values >0.05 were excluded from the model, and first order variables were excluded from the model if they had no significant second order terms, and had a *p*-value >0.05. Explanatory variables were tested for multicollinearity using the variance inflation factor (VIF) and removed for VIF > 10. Residuals plotted against fitted values as well as theoretical quantiles showed approximately normally distributed residuals. F-tests validated the fit of the regression models, and R^∧^2 measured the predictive power.

To account for the effect of the interaction term present in the admission FIM regression model, partial derivatives (also referred to as average marginal effects; Leeper, 2018) with respect to each prediction term were calculated using the margins package in R while controlling for diabetic status. The average marginal effect represents the mean of partial derivatives calculated across different sample values provided to the regression model. To parse out any potential crossover effects with the diabetic status variable, we calculated the marginal effects for diabetic and non-diabetic patients.

## Results

**Figure 2:**
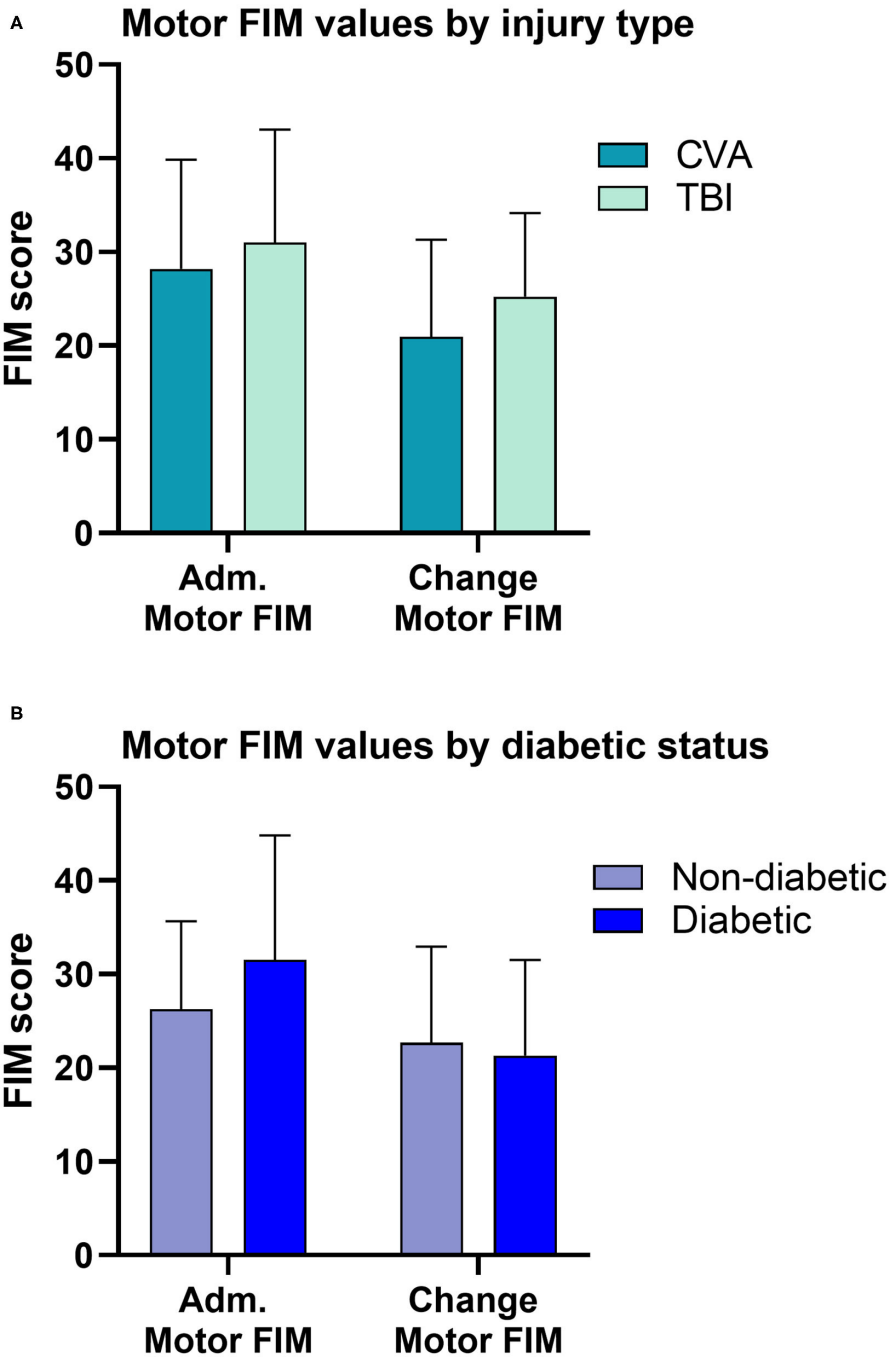
Comparisons of motor FIM values. **(A)** Patients in the CVA group had similar admission and change in motor FIM compared to patients in the TBI group. **(B)** Diabetic status alone, did not influence the admission or the change in motor FIM score. However, diabetic status interacts with the time to start rehabilitation influencing motor FIM (see Table 3).

The matching algorithm yielded a final sample size of 36 patients. Demographic characteristics and clinical measures for the patients are summarized by injury type (TBI vs. CVA) in Table 1, and diabetic status in Table 2. The TBI and CVA patients showed no significant differences in demographic and clinical measures. Specifically the admission motor FIM scores and the change in motor FIM were similar for patients with a CVA and TBI (Figure 2A). The diabetic population was found to be significantly older than the non-diabetic population (65.06 vs. 49.17; *p* = 0.01). However, the admission and motor FIM scores were similar between diabetic and non-diabetic status (Figure 2B). The diabetic population took significantly fewer days from injury to the start of rehabilitation (15.06 vs. 36.5; *p* = 0.03). Significant zero-order Pearson correlations were identified for seven pairs of metrics: admission motor FIM score, motor FIM change, diabetic status, sex (male), patient age, days to start rehabilitation, type of injury (TBI), GCS and total number of steps taken (See Table 3).

**Table 1:** Patient cohort characteristics subdivided by injury type.

Variables^*^	**CVA** ***n* = 27 (75%)**	**TBI** ***n* = 9 (25%)**	***p***
**Diabetic, n (%)**	15 (56%)	3 (33%)	0.44
**Sex, n Male (%)**	22 (81%)	9 (100%)	0.40
Length of Stay (SD)	24.67 (11.26)	19.33 (4.87)	0.06
**Age in years, mean (SD)**	60.93 (13.27)	45.67 (28.74)	0.16
**Days to Start Rehab, mean (SD)**	23.11 (28.72)	33.78 (33.74)	0.41
**Admission motor FIM score, mean (SD)**	28.19 (11.65)	31 (12.05)	0.55
**Motor FIM change, mean (SD)**	20.93 (10.4)	25.22 (8.91)	0.25
**Glasgow Coma Scale, mean (SD)**	13.11 (3.77)	11.11 (4.88)	0.28
RE Sessions (SD)	4.63 (3.76)	3.89 (1.9)	0.45
**Total number of steps taken, mean (SD)**	1084.74 (1110.82)	1170 (1017.47)	0.83

* *Bolded variables represent clinically significant measures used in the regression analyses*.

**Table 2:** Patient cohort characteristics subdivided by diabetes status.

Variables^*^	**Non-diabetic** ***n* = 18 (50%)**	**Diabetic** ***n* = 18 (50%)**	***p***
**TBI, n (%)**	6 (33%)	3 (17%)	0.44
**Sex, n Male (%)**	14 (78%)	17 (94%)	0.34
Length of Stay (SD)	25.22 (11.75)	21.44 (21.44)	0.28
**Age in years, mean (SD)**	49.17 (22.37)	65.06 (10.79)	0.01
**Days to Start Rehab, mean (SD)**	36.5 (38.34)	15.06 (11.35)	0.03
**Admission motor FIM score, mean (SD)**	26.28 (9.35)	31.5 (13.31)	0.18
**Motor FIM change, mean (SD)**	22.67 (10.26)	21.33 (10.19)	0.70
**Glasgow Coma Scale, mean (SD)**	11.44 (4.84)	13.78 (2.86)	0.09
RE Sessions (SD)	4.5 (2.5)	4.39 (4.16)	0.92
**Total number of steps taken, mean (SD)**	1263.22 (883.42)	948.89 (1242.77)	0.38

* *Bolded variables represent clinically significant measures used in the regression analyses*.

**Table 3:** Pearson correlations for various metrics and parameters considered to affect rehabilitation outcome.

	**Admission motor FIM score**	**Motor FIM change**	**Diabetic status**	**Sex (Male)**	**Patient age**	**Days to Start Rehab**	**TBI**	**GCS**	**Total num. steps taken**
Admission motor FIM score	1	0.12	0.23	0.26	0.37^*^	−0.34^*^	0.11	0.15	−0.27
Motor FIM change		1	−0.07	0.06	−0.2	0.03	0.19	−0.09	0.3
Diabetic status			1	0.24	0.42^*^	−0.36^*^	−0.19	0.29	−0.15
Sex (Male)				1	0.25	−0.26	0.23	−0.22	−0.1
Patient age					1	−0.56^*^	−0.35^*^	0.44^*^	−0.29
Days to Start Rehab						1	0.16	−0.24	0.29
TBI^∧^							1	−0.21	0.03
GCS								1	0.04
Total num. steps taken									1

* *Represents significant correlation between factors p < 0.05. ^∧^Represents the impact of having a TBI relative to a CVA*.

The multivariate linear regression assessed predictors for discharge motor FIM and found admission motor FIM score and total robotic steps to be statistically significant predictors. For each point scored higher on the admission motor FIM, the discharge FIM was found to increase by 1.19 (*p* < 0.001; 95% CI 0.89–1.49). For every 1,000 steps taken in the RE, a three-point increase in discharge motor FIM was found (*p* < 0.04; 95% CI 0.18–6.6). Our regression modeled 65% of the variance (adj *R*^2^ = 0.62) and was found to be statistically significant [*F*_(2,33)_ = 33.41; *p* < 0.001].

A second multivariate linear regression modeled predictors for admission motor FIM score. The days from injury to the start of rehabilitation, diabetic status, as well as the interaction term for these variables were found to be statistically significant [*F*_(3,32)_ = 4.17; *p* = 0.008]. After computing the partial derivatives and controlling for diabetic status, we found waiting an additional day to start rehabilitation within the diabetic cohort correlated with a −0.71 point drop in admission motor FIM score (*p* = 0.001; 95% CI −1.14–0.29). We found no significant correlation for diabetic status, nor days to start rehabilitation within the non-diabetic cohort. Our findings suggest that waiting 10 days to start rehabilitation as a diabetic patient, even when using a RE in the rehabilitation process, will correlate with a 7.1 point drop in admission FIM score.

## Discussion

We present a comparative analysis amongst patients with a history of CVA and TBI, showing similar post RE rehabilitation outcomes regardless of the injury type. Both CVA and TBI are non-progressive central nervous system condition that have very similar impairments (DiRocco, 1995). Because of the similarity in impairment between CVA and TBI, the use of RE for TBI patients was conceivable. While it is recognized that additional controlled clinical trials are needed for exoskeleton rehabilitation in TBI, this preliminary study illustrates similar, positive outcomes in functionality to those patients treated with the exoskeleton which had a CVA. To our knowledge, this is the first study reporting diabetic status, time to rehabilitation and steps taken in the exoskeleton as predictive factors of functional outcomes in a cohort of Hispanic patients. This study is one of the first showing that the type of acquired brain injury is not a determinant of motor functional outcome after RE use.

Patients within the TBI group were significantly younger than patients in the CVA group. There are several possible explanations for this observation. First, more than 80% of all CVA incidences across the nation occur in people 65 years and older (Blackwell and Villarroel, 2018), in comparison to TBI for which two of the high risk groups are young children (age 0 to 4) and older adolescents (age 15–19; Centers for Disease Control and Prevention, 2015). Second, the use of RE required specific physical (e.g., height and weight) characteristics as well as physiological (e.g., bone density) to prevent injuries. It is well-documented that older patients (>50 years of age) are at higher risk of osteoporosis (Lane, 2006), which is one of the main contraindications for the use of the exoskeleton, excluding them as potential users. Patient-preference is held at the highest priority when identifying eligible users. Anecdotal evidence from our hospital suggests that older patients are less likely to prefer RE rehabilitation, while younger patients are more eager to engage in the use of bionics and robotics, which could partially explain the age difference in our study cohort. Additional studies are needed to explore the generational gap in rehabilitation preferences, especially for the use of robotic devices.

Patients who suffer TBI, even when mild, could end up having impairments up to 1 year post injury (Nelson et al., 2019). Cognitive impairments after TBI could have even a longer sequelae, lasting up to 4 years, as previously reported (Theadom et al., 2019). The sequelae post-TBI that affects cognitive function is known as post-concussive syndrome (Permenter et al., 2020). To minimize its impact in patient's quality of life, rehabilitation approaches that promote neuroplastic changes in the brain, such as those produced by exoskeleton training, could result in an overall increase in functional domains for the patient after a TBI or CVA (Berger et al., 2019). While the overall improvement in cognitive FIM scores in the current cohort of patients was small, a 1 point increase in FIM scores corresponds to an increase of 1.08 more likely to be discharged to the home rather than to institutionalized care (Thorpe et al., 2018); hence decreasing healthcare cost on the long-term.

The use of robotic interventions in patients with TBI was approved by the FDA at a later time point than for CVA. This delay had in part contributed to a reduced number of studies using RE for TBI. A systematic review from 2011 reported 10 randomized controlled trials for the use of robotic devices in CVA rehabilitation, but none for TBI (Tomida et al., 2019). However, recent reports indicate increased use of robotic rehabilitation following TBI; for example, to quantify the degree of impairment (Logan et al., 2018), improve cognitive function (Maggio et al., 2020), to support treadmill training (Esquenazi et al., 2013), to measure brain activity while having robotic gait training (Lapitskaya et al., 2011), and to increase gait velocity (Esquenazi et al., 2017). While the outcomes of these studies are mixed due to the differences in patient population, training techniques used, and type of equipment, it is promising that the field is recognizing how robotics present with a novel opportunity to improve quality of life in patients with TBI.

A recent study exploring the longitudinal effects of TBI revealed that higher total health burden was associated with poorer functional motor and cognitive trajectories (Kumar et al., 2020). To decrease the negative impact that TBI might have, it is hypothesized the continuous use of RE as a rehabilitation device in our hospital will significantly continue to improve functional outcomes. Since the intervention with RE revealed similar outcomes to the CVA patient cohort, even with small sample size, it is foreseen that many more patients eligible to use exoskeleton rehabilitation devices will greatly benefit from this therapeutic modality.

Diabetes is a modifiable factor strongly associated with CVA of both the ischemic and hemorrhagic type (O'Donnell et al., 2010; Lee et al., 2012). Unfortunately, the risk for CVA in diabetic patients is higher in the younger population, specifically before the age of 55 years (Khoury et al., 2013). In addition, people with diabetes also tend to suffer from other comorbidities such as hypertension and high cholesterol. A previous review on the topic suggested that stroke outcomes in individuals with diabetes depend on a prompt and persistent implementation of therapies, for the successful recovery of the individual (Chen et al., 2017). When it comes to TBI, diabetes is a well-known complication of patients in the intensive care units, and is predictive of increased mortality and poorer outcomes (Gempeler et al., 2020). Additionally, detrimental effects of pre-existing diabetes have been reported in TBI using an animal model, showing increased brain contusion volume and higher inflammatory markers (Tatara et al., 2020). In light of these previous findings, we propose that including RE rehabilitation for patients who suffer either TBI or CVA in a prompt fashion, could provide increased benefits when done in parallel with strict glucose control measures.

### Study Limitations

The literature on the use of RE devices for TBI resoundingly contains the limitation of sample size (Dijkers et al., 2019), resulting limited study power. We recognize that the current study has this inherent limitation of sample size, but even small studies with a less than perfect experimental design will help other researchers and clinicians in the field to make informed decisions. The limitation in the selection of a statistically significant sample size corresponds to the fact that patients need to meet a specific set of criteria to be eligible for rehabilitation with exoskeleton devices, as outlined in the methods section. It is also important to recognize that our study is based on a retrospective sample of patients, most of which were of Hispanic origin; thus, generalization to other ethnic groups might be limited. While individual comorbidities may not directly affect outcomes observed in the use of exoskeleton, chronic conditions such as pressure sores, spinal instability, deep vein thrombosis, uncontrolled hypertension, among others (Palermo et al., 2017) are direct contraindications for the use of the device; hence limiting the pool of patients that can be included in the research studies. Well-controlled randomized trials on exoskeleton rehabilitation are emerging as per published protocols (Louie et al., 2020). We recognize that the follow up of the patients beyond their discharge from the rehabilitation hospital was not done. However, our goal was to observe the short-term effects of the RE in the FIMS of patients since many other factors could influence the long-term outcomes of patients once discharge (care at home, other comorbidities, etc.). Future prospective studies should design for collection of long term (6 months−1 year) outcomes after RE.

### Conclusions

Physical therapists are more frequently employing robotic technology to enhance traditional rehabilitation therapy methods. The use of robotic technology to more specifically and deliberately treat patients with brain injury is allowing for increased intensity and duration of activity, and engagement of the patient with an activity of interest. This study demonstrated that gait training with a RE in patients with TBI and CVA leads to similar improvements on FIM scores. The present analysis also demonstrated that key clinic-biologic factors including presence or absence of diabetes, together with start to rehabilitation play key roles in discharge FIM scores. Large multicenter randomized controlled trials comparing the use of the exoskeletons vs. traditional methods in the TBI population are warranted to verify and expand the preliminary findings of this study. Findings lead us to hypothesize that encouraging patients to take a maximum number of steps while using the RE, will lead to increased FIM motor scores. Our study also highlights the need for specific attention into the diabetic patient population such as a need for swift admission into a comprehensive rehabilitation program that includes the option of RE rehabilitation. Taken together, our findings support previous findings showing that the use of RE enhances dosing during motor rehabilitation (Nolan et al., 2020), and that early interventions may produce the most benefit to the recovery of patients.

## Data Availability Statement

The raw data supporting the conclusions of this article will be made available by the authors, without undue reservation.

## Ethics Statement

The studies involving human participants were reviewed and approved by DHR Health Institute for Research and Development. Written informed consent for participation was not required for this study in accordance with the national legislation and the institutional requirements.

## Author Contributions

LT and MA conceptualized the original research idea and wrote the original research protocol. AM collected and verified all data used in the project. PR analyzed the data and prepared figures, methods, and results sections. AT-R supervised data analysis and wrote the first version of the manuscript with LT. All authors contributed to the final edits of the document and approved the current version.

## Conflict of Interest

The authors declare that the research was conducted in the absence of any commercial or financial relationships that could be construed as a potential conflict of interest.
